# Angiopoietin-Like Growth Factor Involved in Leptin Signaling in the Hypothalamus

**DOI:** 10.3390/ijms22073443

**Published:** 2021-03-26

**Authors:** Yunseon Jang, Jun Young Heo, Min Joung Lee, Jiebo Zhu, Changjun Seo, Da Hyun Go, Sung Kyung Yoon, Date Yukari, Yuichi Oike, Jong-Woo Sohn, Minho Shong, Gi Ryang Kweon

**Affiliations:** 1 Department of Biochemistry, Chungnam National University School of Medicine, Daejeon 35015, Korea; yunseonj@cnu.ac.kr (Y.J.); junyoung3@gmaiil.com (J.Y.H.); rmj1102@cnu.ac.kr (M.J.L.); zhujiebo2019@o.cnu.ac.kr (J.Z.); justin20@o.cnu.ac.kr (C.S.); goda1046@o.cnu.ac.kr (D.H.G.); 201950879@o.cnu.ac.kr (S.K.Y.); 2 Department of Medical Science, Chungnam National University School of Medicine, Daejeon 35015, Korea; 3 Infection Control Convergence Research Center, Chungnam National University School of Medicine, Daejeon 35015, Korea; 4 Frontier Science Research Center, University of Miyazaki, Miyazaki 889-1692, Japan; dateyuka@med.miyazaki-u.ac.jp; 5 Department of Molecular Genetics, Graduate School of Medical Sciences, Kumamoto University, Kumamoto 860-8556, Japan; oike@gpo.kumamoto-u.ac.jp; 6 Department of Biological Sciences, Korea Advanced Institute of Science and Technology, Daejeon 34141, Korea; jwsohn@kaist.ac.kr; 7 Department of Internal Medicine, Chungnam National University School of Medicine, Daejeon 35015, Korea

**Keywords:** hypothalamus, AGF, leptin, POMC neuron

## Abstract

The hypothalamic regulation of appetite governs whole-body energy balance. Satiety is regulated by endocrine factors including leptin, and impaired leptin signaling is associated with obesity. Despite the anorectic effect of leptin through the regulation of the hypothalamic feeding circuit, a distinct downstream mediator of leptin signaling in neuron remains unclear. Angiopoietin-like growth factor (AGF) is a peripheral activator of energy expenditure and antagonizes obesity. However, the regulation of AGF expression in brain and localization to mediate anorectic signaling is unknown. Here, we demonstrated that AGF is expressed in proopiomelanocortin (POMC)-expressing neurons located in the arcuate nucleus (ARC) of the hypothalamus. Unlike other brain regions, hypothalamic AGF expression is stimulated by leptin-induced signal transducers and activators of transcription 3 (STAT3) phosphorylation. In addition, leptin treatment to hypothalamic N1 cells significantly enhanced the promoter activity of AGF. This induction was abolished by the pretreatment of ruxolitinib, a leptin signaling inhibitor. These results indicate that hypothalamic AGF expression is induced by leptin and colocalized to POMC neurons.

## 1. Introduction

The hypothalamus controls feeding behavior for the maintenance of whole-body energy balance, and dysregulation of energy intake leads to obesity [1]. Leptin is an anorectic hormone to regulate the hypothalamic neural network including proopiomelanocortin (POMC) neurons, producing α- melanocyte-stimulating hormone (MSH) [2]. Insensitivity to leptin signaling is observed in obese patients, despite the increase in leptin produced by adipose tissue [3,4]. It is reported that the combination of recombinant human leptin, metreleptin and pramlintide enhanced the reduction of body weight in a clinical trial [5]. Additionally, celastrol, originated from thunder god vine roots, increased brain leptin sensitivity to promote weight loss and food intake [6]. However, while considerable research on the beneficial effect of leptin on feeding control and energy expenditure has been done, downstream molecules of leptin signaling in the hypothalamic neuron still remain elusive. 

Hypothalamic leptin signaling initiates Janus kinase 2-signal transducers and activators of transcription 3 (JAK2-STAT3) signaling and extracellular signal-regulated kinase (ERK) to regulate food intake [7] and adenosine monophosphate-activated protein kinase (AMPK) signaling for energy homeostasis [8]. The POMC neuronal population is located within the arcuate nucleus (ARC) of the hypothalamus. In postprandial state, a POMC neuron produces an anorectic molecule on leptin binding to leptin receptor (LEP-R) [2]. Leptin promotes POMC expression through the phosphorylation of STAT3. A significant increase in body weight was observed in *Pomc*-Cre, *Lepr^flox/flox^* mice, in which the leptin receptor was deleted in the POMC neuron [9]. Although leptin signaling in the POMC neuron is important for the regulation of appetite, the mediator of leptin signaling in the POMC neuron is only partially identified. In the current study, we identified angiopoietin-like growth factor (AGF) as a downstream of leptin, and AGF expression is enhanced by leptin in the hypothalamus.

AGF has been identified as a peripheral activator of energy expenditure [10,11] that antagonizes obesity and insulin resistance. Whole-body AGF-knockout (KO) mice exhibited severe obesity [12,13]. Adenovirus-mediated hepatic overexpression of AGF showed to reverse the obesity of AGF-KO mice [12]. AGF is a member of the angiopoietin-like proteins (ANGPTLs) family composed of eight members, all of which possess a coiled-coil domain at the N-terminus and a fibrinogen-like domain at the C-terminus [14]. ANGPTLs are orphan ligands that do not bind Tie1 (tyrosine kinase with immunoglobulin-like and epidermal growth factor (EGF)-like domains 1) or Tie2 receptors—targets of angiopoietin [14]. However, the existence of AGF and the regulating factor of AGF in brain is unknown. In the present study, we demonstrate that AGF is induced by the leptin hormone in the hypothalamus and expressed in POMC neurons.

## 2. Results

### 2.1. Angptl6 Is Expressed in the Hypothalamus and Induced after Feeding

**Figure 1:**
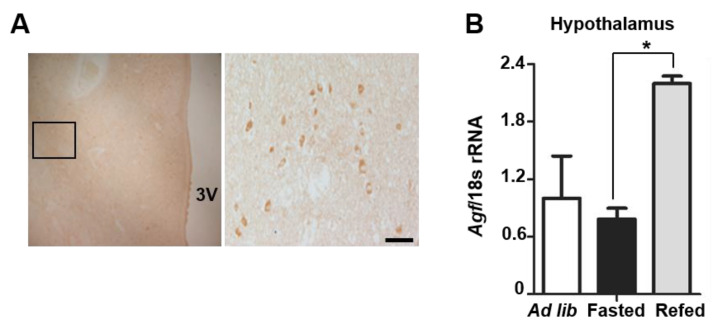
Angiopoietin-like growth factor (AGF) is expressed in the hypothalamus. (**A**) Immunohistochemical detection of AGF in the human brain. The boxed area within the image indicates a magnified field. (**B**) *Agf* mRNA expression in the hypothalamus of *ad libitum-fed*, overnight fasted or refed mice, confirmed by qPCR analysis (*n* = 5/group). Data represent means ± SEM (* *p* < 0.05). Scale bars: 50 μm.

Hypothalamic appetite control is critical for the maintenance of whole-body energy balance, and the impairment of anorectic signaling is associated with obesity [1]. AGF is known as an activator of energy expenditure in the liver and adipocytes [12,13]. Although AGF protein expression and function in the hypothalamus have not been investigated, AGF mRNA has been detected in the brain of C57BL/6 mice [15]. To determine whether AGF is expressed in the hypothalamus, we performed immunohistochemistry on human brain. As shown in Figure 1A, AGF immunoreactivity was detected in the hypothalamus of postmortem human brain. Because the hypothalamus is sensitive to nutritional status and governs whole-body energy homeostasis [1,2,16,17], we tested whether hypothalamic AGF expression is affected by changes in nutritional status. Mice were allowed to freely approach food, and the refed group was fed after overnight fasting. Fasting did not alter AGF mRNA level in the hypothalamus or epididymal white adipose tissue (eWAT), but did decrease hepatic AGF mRNA level (Figure S1A,B). In contrast, AGF mRNA level increased ~2-fold in the hypothalamus after refeeding (Figure 1B) and modestly increased in liver and eWAT, indicating that feeding induces AGF expression in metabolic organs. Fasting and refeeding status of mice were confirmed by measurements of blood glucose level and insulin concentration in plasma (Figure S1C,D). 

### 2.2. Angptl6 Is Expressed in POMC Neurons of the Hypothalamus

**Figure 2:**
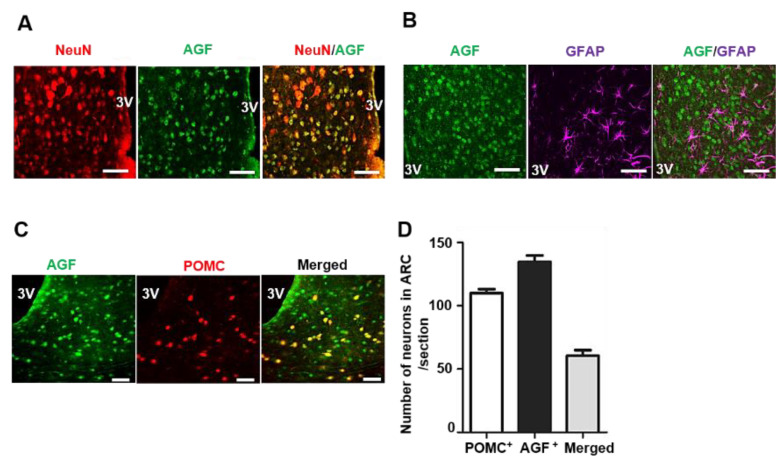
AGF is colocalized to proopiomelanocortin (POMC) neurons rather than glial fibrillary acidic protein (GFAP)-positive cells in the hypothalamus. (**A**,**B**) Immunofluorescence staining of AGF (green) and NeuN (red), a neuronal nuclei marker and GFAP (magenta), an astrocyte marker in the hypothalamus section. (**C**) POMC neurons in the hypothalamus, detected based on red fluorescence in POMC-specific tandem dimer Tomato (tdTomato) expressing reporter mice, and AGF immunofluorescence staining. Brain slices were prepared by cryosectioning (25 μm/section). Sections were incubated overnight at 4 °C with Alexa 488-conjugated mouse anti-AGF antibody (1:100). Fluorescence was visualized by confocal microscopy. A yellow-colored neuron represents the colocalization of AGF with POMC. (**D**) Number of AGF and POMC double-positive neurons in the ARC (between −1.7 and −3.4 mm from bregma) presented as means ± SD (*n* = 10/slide). Scale bars: 50 μm (upper).

The hypothalamus delivers pivotal anorectic signaling after feeding. Because AGF-positive cells were found in the human hypothalamus (Figure 1A), we performed immunofluorescence staining of AGF in mice to identify the localization and cell type in the hypothalamus. As shown in Figure 2A, AGF immunoreactivity was detected in the ARC where the feeding regulatory circuit is localized. AGF immunoreactivity exhibited a 91% overlap with the neuronal marker, NeuN. However, AGF did not colocalize with glial fibrillary acidic protein (GFAP) (Figure 2B), one of a marker of astrocytes (a type of glial cell). Leptin binds to the LEP-R of the POMC-expressing neuron, which is located in the ARC of the hypothalamus and activates anorectic signaling in a postprandial state [18,19]. POMC activation reduces food intake through the release of α-MSH, and *Pomc*-deficient mice in the ARC (Arc*Pomc*^−/^^−^) showed obesity and hyperphagia [20]. Because AGF in the hypothalamus was induced by feeding (Figure 1B), we verified the spatial expression of AGF in relation to these major appetite-controlling neurons by performing immunofluorescence staining for AGF in the brain of POMC-mCherry reporter mice. We found that 45.0% of AGF-positive neurons in the ARC of the hypothalamus were also POMC-positive (Figure 2C,D). These results indicate that AGF localized to the POMC-expressing neuronal population and it is not colocalized to GFAP-positive cells.

### 2.3. AGF Expression Is Associated with Leptin-Induced STAT3 Phosphorylation

**Figure 3:**
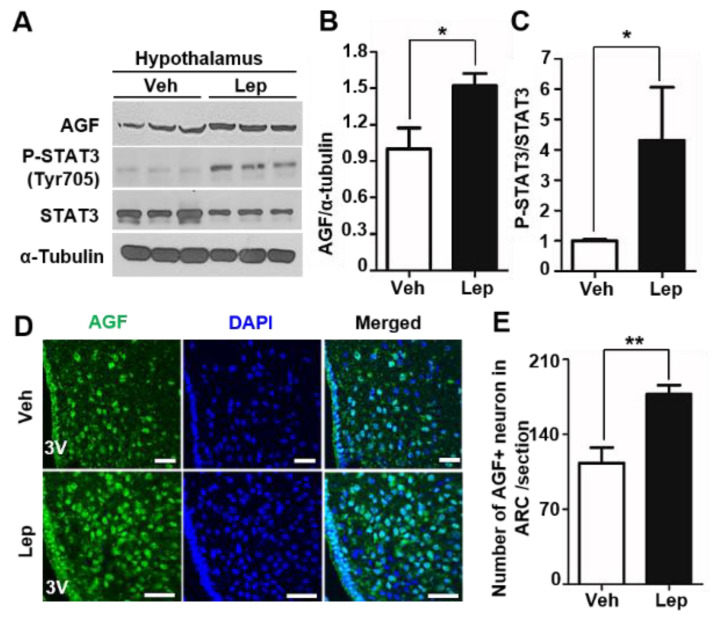
AGF expression is induced by leptin-stimulated signal transducer and activator of transcription 3 (STAT3) activation. (**A**) AGF level in the hypothalamus of C57BL/6 mice after intraperitoneal injection of recombinant mouse leptin (3 mg/kg) or vehicle, determined by Western blotting. AGF, pSTAT3 (Tyr705), STAT3 and α-tubulin were detected in tissue lysates containing equal amounts of protein from the hypothalamus of each group. (**B**,**C**) Band intensities of AGF and pSTAT3 measured by the ImageJ program; values are normalized to vehicle-injected samples. (**D**) AGF and DAPI (4′,6-diamidino-2-phenylindole) co-staining in brain sections from leptin- or vehicle-injected mice. Immunoreactivity was detected by confocal microscopy. (**E**) Number of AGF-positive neurons in the ARC. Data represent means ± SEM (* *p* < 0.05, ** *p* < 0.01).

Insulin and leptin, which are secreted from peripheral organs and act on the hypothalamus, are induced during the postprandial state and serve to inhibit food intake [21,22]. Accordingly, we tested the possibility that this hormone might act through effects on AGF level to modulate feeding behavior. Treatment of the insulin receptor-expressing cell lines, SN4741 and C2C12, with insulin (100 nM) had no effect on AGF protein or mRNA level (Figure S2A–C). Next, we examined the relationship between AGF and leptin, the latter of which dominates feeding behavior through the activation of anorectic signaling in the hypothalamus [18,23]. The leptin receptor (LepRb) is expressed in hypothalamic neurons, and, upon binding leptin, induces the JAK2 (Janus kinase 2)-mediated phosphorylation of STAT3 (signal transducer and activator of transcription 3) [18,19,24]. To examine changes in AGF level induced by leptin in vivo, we injected mice intraperitoneally with leptin (3 mg/kg) and sacrificed mice after 1 h. Leptin induced an increase in AGF protein in the hypothalamus (Figure 3A,B), but not in other brain areas, including the cortex, striatum and midbrain (Figure S3A). Immunostaining for Tyr705–phosphorylated STAT3 (pSTAT3), a leptin signaling marker, was increased in hypothalamus tissue (Figure 3A,C) and the ARC by leptin injection (Figure S3B). To demonstrate the response of AGF-expressing neurons to leptin, we immunostained for AGF in the ARC of mice injected with leptin or vehicle (Figure 3D). This analysis showed that leptin increased the number of AGF-positive neurons by 56% (Figure 3D,E). Collectively, these data suggest that leptin induces hypothalamic AGF level.

### 2.4. AGF Promoter Activity Is Enhanced by Leptin

**Figure 4:**
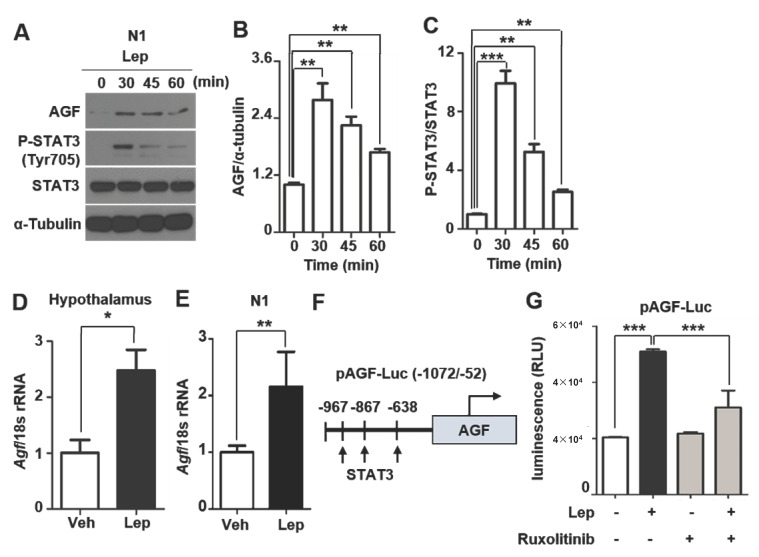
AGF promoter activity is increased by leptin. (**A**) AGF protein level in N1 cells, determined after treatment with 100 ng/ml leptin for 0, 30, 45 and 60 min. (**B**,**C**) AGF and pSTAT3 band intensities, measured using the ImageJ program. (**D**,**E**) AGF mRNA expression level in the hypothalamus (**D**) and in N1 cells (**E**) after leptin administration, normalized to 18s rRNA level, as determined by qPCR (*n* = 5/group). (**F**) Location of STAT3 binding sequences in the AGF promoter (−1072 to −52 bp). (**G**) AGF promoter activity, measured in N1 cells transfected with an AGF promoter-luciferase reporter plasmid after a 45 min incubation with leptin (100 ng/ml). Cells were pre-incubated in the presence or absence of the JAK2 inhibitor, ruxolitinib (1 μM) for 1 h. N1 cells were harvested and luminescence was measured. Data represent means ± SEM (* *p* < 0.05, ** *p* < 0.01, *** *p* < 0.001).

Consistent with in vivo experiments, both AGF and pSTAT3 expression were increased after leptin treatment in N1 cells (Figure 4A–C). We further found that AGF mRNA level was 2.5-fold higher in the hypothalamus of leptin-injected mice (Figure 4D) and 2-fold higher in leptin-treated N1 cells compared with vehicle-treated controls (Figure 4E), suggesting that leptin induces AGF transcription. To test this, we first sought to identify transcription factor binding sites in the AGF promoter using an online search program (http://tfbind.hgc.jp/ accessed on 23 May 2020), which predicted three consensus STAT3 binding sequences in a 1020-bp (−1072 to −52 bp) 5′ untranslated region (Figure 4F). Using a promoter-reporter construct in which this 1020-bp region was placed upstream of the luciferase gene, we tested whether the promoter activity of AGF was affected by leptin treatment. In N1 cells transfected with this promoter-reporter construct, leptin (100 ng/ml) induced a 2.6-fold increase in reporter activity within 45 min (Figure 4G). Notably, induction of AGF promoter activity by exogenous leptin treatment was ablated by a 1 h pre-incubation with ruxolitinib (1 μM), a leptin signaling inhibitor that blocks JAK2-mediated phosphorylation (Figure 4G). These results indicate that AGF transcription is induced by leptin and mediated by phospho-STAT3, a downstream of leptin/JAK2 signaling.

## 3. Discussion

Modulation of appetite by the hypothalamus is pivotal for energy homeostasis and is associated with the regulation of body weight [1,2,17]. Leptin binding to LEP-R on POMC neurons provokes anorectic signaling in the hypothalamus. It is reported that leptin activates ERK1/2 in the ARC for anorectic and sympathetic effect as confirmed by leptin-induced reduction of food intake was reversed by ERK inhibitor U0126 injection [7,8]. However, the downstream mediator of leptin signaling to modulate hypothalamic neuronal activity that is associated with appetite control is still elusive. 

AGF, a known peripheral activator of energy expenditure, is determined by leptin in mice hepatocyte, and serum AGF level is paradoxically increased in human patients with metabolic disease due to compensation [25]. In the previous report, whole-body AGF KO mice showed extreme obesity due to a decrease in energy consumption with no change in food intake. The adenovirus-mediated reconstitution of AGF leads to a decrease in body weight and improved glucose tolerance [12], suggesting that peripheral AGF has a role in energy expenditure. However, alteration of brain AGF expression in this model was not examined and cell-type-specific AGF expression in the hypothalamus was unknown. In the current study, we address the fact that AGF is expressed in the hypothalamus and has leptin responsiveness. As we observed that AGF promoter activity was enhanced by the treatment of leptin and ablated this effect by ruxolitinib, JAK1/2 inhibitor in hypothalamic N1 cells (Fig. 4G), it would be supportive to elucidate the involvement of AGF in leptin signaling if reproducible effects similar with cells occur in the hypothalamus of mice. 

To assess whether AGF expression is dependent on leptin in the hypothalamus, *ob/ob* and *db/db* mice with leptin or leptin receptor deficiency could be useful. There is a possibility that AGF expression is reduced in ob/ob mice and exogenous leptin may restore AGF level in the hypothalamus. Additionally, feeding-induced hypothalamic increase in AGF expression may be ablated in these mice. Hypothalamic control of feeding by leptin is disrupted in obese subjects due to insensitivity to leptin signaling, despite the elevated level of circulating leptin [26,27,28]. The alteration of hypothalamic AGF in obesity remains to be elucidated.

AGF immunoreactivity was detectable in the hypothalamus of human postmortem brain around the third ventricle. Neuronal cell type and nucleus where AGF is localized in the human hypothalamus was not identified. The human protein atlas program (https://www.proteinatlas.org/, accessed on 23 May 2020) revealed AGF expression in the hypothalamus, but it does not present specific regions such as the paraventricular nucleus (PVN) and ARC. In the case of mice, AGF immunoreactivity predominantly colocalized to POMC neurons in the ARC (Figure 1A and Figure 2C), not GFAP-positive cells. AGF expression was not restricted to the ARC but observed in dorsomedial hypothalamic nucleus (DMH) and PVN (data not shown). It is known that leptin administration activates NUCB2/nesfatin-1-expressing neurons in PVN to inhibit food intake [29]. Additionally, stimulation of the Thyrotropin-releasing hormone (TRH) neuron by leptin leads to an increase in pSTAT3 and neuronal activation, reducing appetite [30]. Therefore, to determine whether PVN AGF colocalize to neurons related to food intake regulation would give an insight into the role of AGF in appetite control and metabolism. Recently, it has been reported that tanycytes transport leptin into the hypothalamus and it is required for the regulation of normal hypothalamic leptin signaling [31]. To determine distinct neuronal populations which express AGF in the ARC and whether tanycytes co-express AGF could give an insight into AGF as a downstream mediator of leptin signaling.

A physiological role of hypothalamic AGF as a downstream effector of leptin in POMC neurons has not been determined in this study. Based on our finding that 45% of AGF-expressing neurons colocalized with POMC neurons in the ARC, the highly overlapped expression of AGF and POMC gives rise to the possibility that AGF has a role in POMC neuron-mediated metabolic changes such as appetite regulation and energy expenditure, which are promoted by leptin [32]. Both AGF expression and STAT3 phosphorylation were significantly induced after 30 min of leptin treatment. In contrast, pSTAT3 level declined after 60 min, whereas AGF level was sustained (Figure 4A). From this, we infer that, after induction by pSTAT3, AGF could act to regulate leptin downstream signaling, for example, by inducing JAK2 phosphatase, which is a negative regulator of this signaling [33]. It is also implied that leptin-induced AGF interacts with leptin signaling pathways such as ERK and AMPK in leptin receptor-expressing neurons to control appetite and energy balance. Further study for verification of the physiological role of hypothalamic AGF is including use of hypothalamic POMC neuron-specific AGF KO mice via assessment of an alteration of leptin signaling and α-MSH level. In addition, recording the neuronal activity of POMC in a condition of increasing AGF expression by leptin treatment using a patch-clamp study could be a useful approach. Our study suggests that AGF is a downstream molecule of leptin signaling in the hypothalamus. 

## 4. Materials and Methods

### 4.1. Animals

Male C57BL/6 mice were purchased from Harlan Teklad (Indianapolis, IN, USA). Mice were maintained at 22 °C under a 12 h light–dark cycle. For diet-induced obesity, 5-week-old C57BL/6 mice were fed a high-fat diet (60% fat; Research Diets Inc., New Brunswick, NJ, USA). Leptin (3 mg/kg, R&D systems, Minneapolis, MN, USA) was dissolved in 0.9% saline and intraperitoneally injected into C57BL/6. Animal experiments were approved by the Institutional Animal Care and Use Committee of Chungnam National University (Ethical approval number, 201903A-CNU-46, approved on 1 June 2019).

### 4.2. Cell Culture

HypoE-N1 mice embryonic hypothalamus cells were maintained in Dulbecco’s Modified Eagle Medium (DMEM) containing 10% fetal bovine serum (Gibco, Waltham, MA, USA), 1% penicillin/streptomycin at 37 °C under 5% CO_2_/21% O_2_ condition.

### 4.3. Human Brain Tissue

The hypothalamus was obtained from postmortem brains of otherwise healthy individuals at the University of Miyazaki, Japan, with the approval of the institutional review board (approval number, C-0037; 11 May 2018). Written informed consent was acquired from each outpatient, and the study was conducted according to provisions of the Declaration of Helsinki. 

### 4.4. Immunofluorescence Staining and Immunohistochemistry

Postmortem hypothalamus was fixed in 4% paraformaldehyde for 24 h, embedded in paraffin, and cut into 4-μm–thick sections. Immunohistochemistry was performed using an anti-human AGF antibody, produced by immunizing rabbits with a synthetic peptide corresponding to amino acids 392–408 (NDKPESTVDRDRDSYSG) of the AGF protein. Sections were pretreated with periodic acid (Nichirei, Tokyo, Japan) and incubated with anti-human AGF antibody (1:100) overnight. Then, the sections were washed with phosphate-buffered saline (PBS) and immunostained using EnVision/horseradish peroxidase (Dako, Glostrup, Denmark). After incubating with 3,3-diaminobenzidine solution, images were acquired using an IX70 microscope (Olympus, Tokyo, Japan). Mice whole brain was dipped in the 4% PFA and moved to 30% sucrose solution. The brain section was cut into 25 μm and blocked for 1 hour with 3% donkey serum (Dako, Glostrup, Denmark) and 0.3% triton x-100. Then, brain sections, including the arcuate nucleus region, were obtained from −1.7 to −3.4 mm from bregma and were incubated with primary antibodies, including a mouse anti-AGF-488 alexa conjugated antibody (1:100, Bioss, MA, USA), anti-NeuN (1:500, Abcam, Cambridge, UK), a chicken anti-GFAP (1:1000, Abcam, Cambridge, UK), and a rabbit anti-POMC (1:300, Phoenix, CA, USA) antibody, overnight at 4 °C. Sections were washed with PBS and incubated in secondary fluorescence antibody. Fluorescence was visualized using a IX70 fluorescent microscope (Olympus, Tokyo, Japan). 

### 4.5. RNA Iolation and Real-Time PCR

The hypothalamus was removed from mice brain and homogenized by TissueLyserII (Qiagen, Netherlands). Total RNA was isolated using Isol-RNA lysis reagent (5 PRIME, South San Francisco, CA, USA). cDNA was synthesized by an moloney murine leukemia virus (M-MLV) reverse transcriptase kit (Invitrogen, Carlsbad, CA, USA). For real-time PCR, after mixing cDNA, primers and 10X SYBR mix, mRNA expression was analyzed using a Rotor Gene 6000 system (Corbett Life Science, Venlo, Netherlands) and normalized to 18s rRNA. Gene-specific primers are listed in Table S1.

### 4.6. Western Blotting

Proteins of mice tissues were prepped and homogenized by TissueLyserII, and N1 cells were extracted using radioimmunoprecipitation assay (RIPA) buffer (1% Nonidet P-40, 0.1% SDS, 150 mM NaCl, 50 mM Tris–HCl pH 7.5 and 0.5% deoxycholate) with 10% of phosphatase and protease inhibitor (Roche, Basel, Switzerland). A total of 12 μg of protein samples was loaded on SDS-PAGE gel and run by electrophoresis; afterwards, it was transferred to polyvinylidene fluoride (PVDF) membrane, blocked by 5% skim milk. Membranes were incubated with primary antibody including a mouse anti-AGF (R&D systems, Minneapolis, MN, USA), a mouse anti-α-tubulin (Santa Cruz Biotechnology, Dallas, TX, USA), anti-phospho-STAT3 and anti-STAT3 (Cell Signaling, Danvers, MA, USA) antibody at 4 °C overnight. Anti-Immunoglobulin G (IgG) horseradish peroxidase antibody (Pierce Biotechnology, MA, USA) corresponds with the host of the primary antibody and was used as a secondary antibody. A protein band was detected by the enhanced chemiluminescence (ECL) system (Thermo Scientific, Waltham, MA, USA) 

### 4.7. Promoter-Luciferase Reporter Assay

N1 cells were grown in 6-well plates and transfected with 1 μg pAGF prom-Luc DNA mixture using lipofectamin reagents (Invitrogen, Carlsbad, CA, USA). The pLightSwitch-prom plasmid encodes RenSP luciferase sequence under control of 1020 bp AGF promoter. After transfection, cells were treated with 100 ng/ml leptin or vehicle for 45 min and harvested for luminiscence reading. Luciferase activity was measured using a Berthold LB9507 luminometer (Berthold Technologies, Black Forrest, Germany).

### 4.8. Statistical Analysis

All data are represented as mean ± standard error mean (SEM) from triplicate results. The statistical analysis was determined using Prizm version 5 software (Graphpad, San Diego, CA, USA). The significance of differences between two groups was analyzed by one-tailed student’s *t*-test. *p* value < 0.05 was considered statistically significant. 

## Data Availability

The datasets generated during and/or analyzed during the current study are available from the corresponding author on reasonable request. No applicable resources were generated or analyzed during the current study.

## Supplementary Materials

The following are available online at https://www.mdpi.com/article/10.3390/ijms22073443/s1, Figure S1: AGF mRNA expression is increased after feeding in mice, Figure S2: AGF expression is not affected by insulin, Figure S3: Leptin injection did not induce AGF expression in various brain region excluding hypothalamus.
