# Supplementary material for: Angiopoietin-Like Growth Factor Involved in Leptin Signaling in the Hypothalamus
Source: Int J Mol Sci. 2021 Mar 26;22(7):3443. doi: 10.3390/ijms22073443 (PMC8037945; doi:10.3390/ijms22073443)
Supplement: Supplementary file 1 [file ijms-22-03443-s001.zip › Part 1_ supplementary figure.docx]

**Supplementary Materials**

**Materials and methods**

**Determination of blood glucose and serum insulin level**

Blood glucose level of C57BL/6 mice freely fed with chow diet, 12 hours fasted or 2 hours after refed was determined by glucometer (i-SENS, Seoul, Korea). Plasma insulin level was measured using mouse ultrasensitive insulin ELISA kit (ALPCO, NH, USA) according to the manufacturer’s instruction.

**
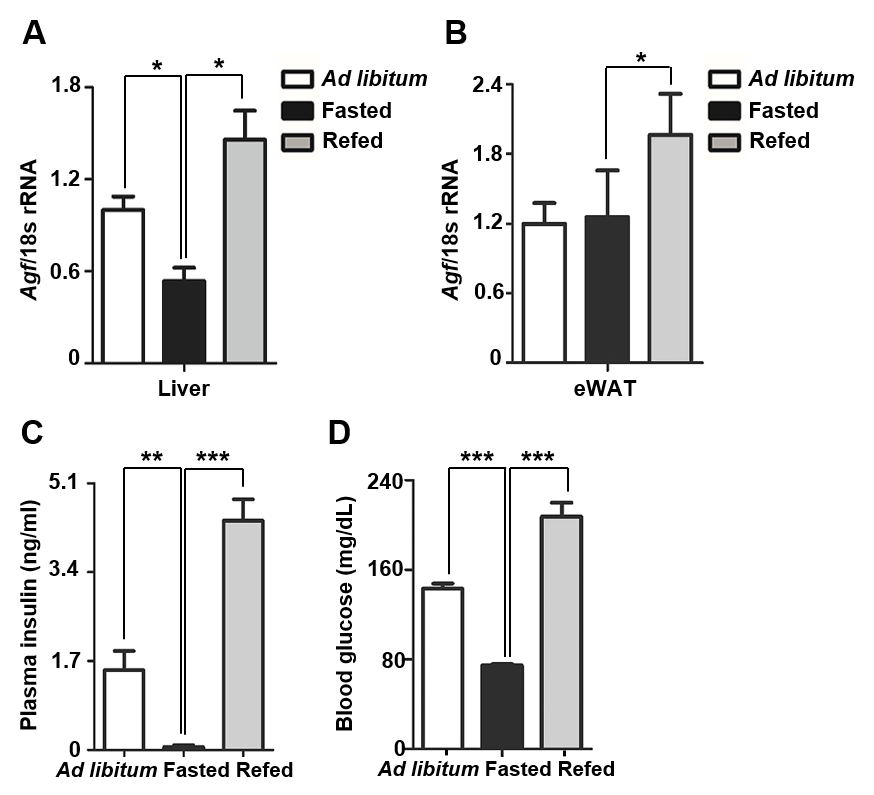
**

**Fig. S1. AGF mRNA expression is increased after feeding in mice.** A, B, AGF level in liver and epididymal white adipose tissue (eWAT) after overnight fasting or refeeding (n=5, each groups). C, D, fasting and feeding was confirmed by measurement of plasma insulin and glucose level. Insulin was measured by ELISA. Data represents as means ± SD. Significant differences: **p*< 0.05, ***p*< 0.01, ****p*< 0.001.


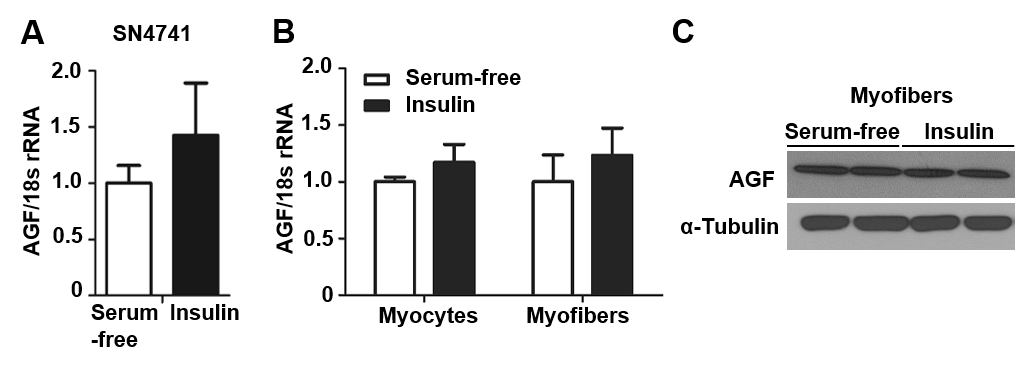


**Fig. S2. AGF expression is not affected by insulin.** A-C, insulin receptor expressing SN4741 cells and C2C12 myocytes and myofibers were treated with 100 nM insulin. Cells were harvested after 1 hour. AGF mRNA level and protein was not altered by insulin treatment.


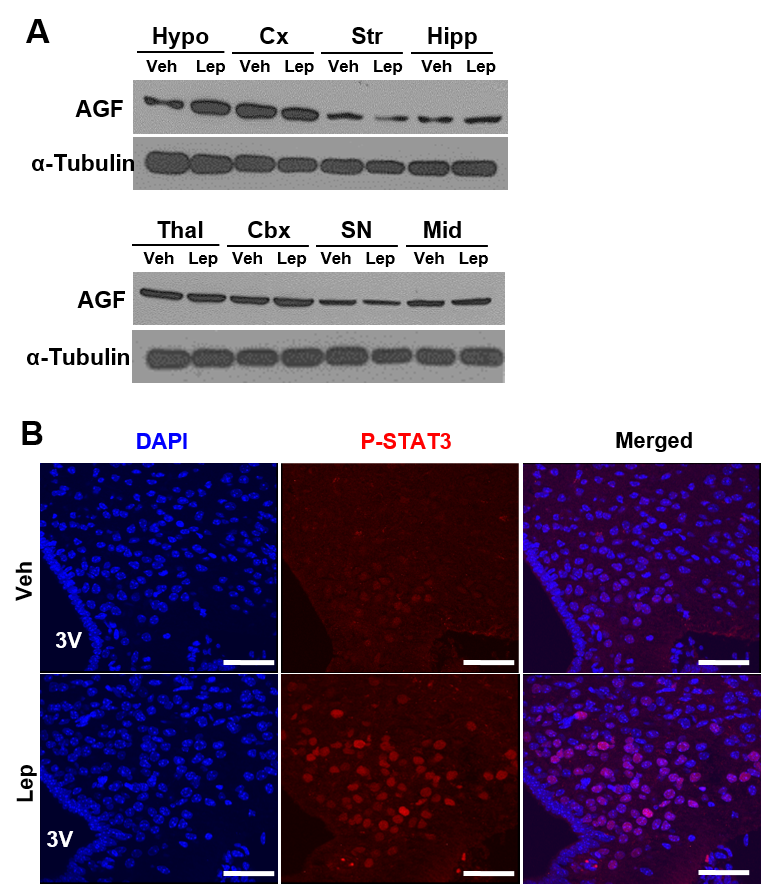


**Fig. S3. Leptin injection did not induce AGF expression in various brain region excluding hypothalamus.** A, AGF protein expression of mice with i.p injection of leptin (3mg/kg) or vehicle was determined by WB analysis using AGF and α-tubulin antibody. B, P-STAT3 and DAPI was co-stained with a specimen of leptin or veh injected brain sections by immunofluorescence staining. Scale bar is 50 µm. Abbreviations: Hypo, hypothalamus; Cx, cerebral cortex; Str, striatum; Hipp, hippocampus; Thal, thalamus; Cbx, cerebellum; SN, substantia nigra; Mid, midbrain.
