# Supplementary figures and images for: Angiopoietin-Like Growth Factor Involved in Leptin Signaling in the Hypothalamus
Source: Int J Mol Sci. 2021 Mar 26;22(7):3443. doi: 10.3390/ijms22073443 (PMC8037945; doi:10.3390/ijms22073443)

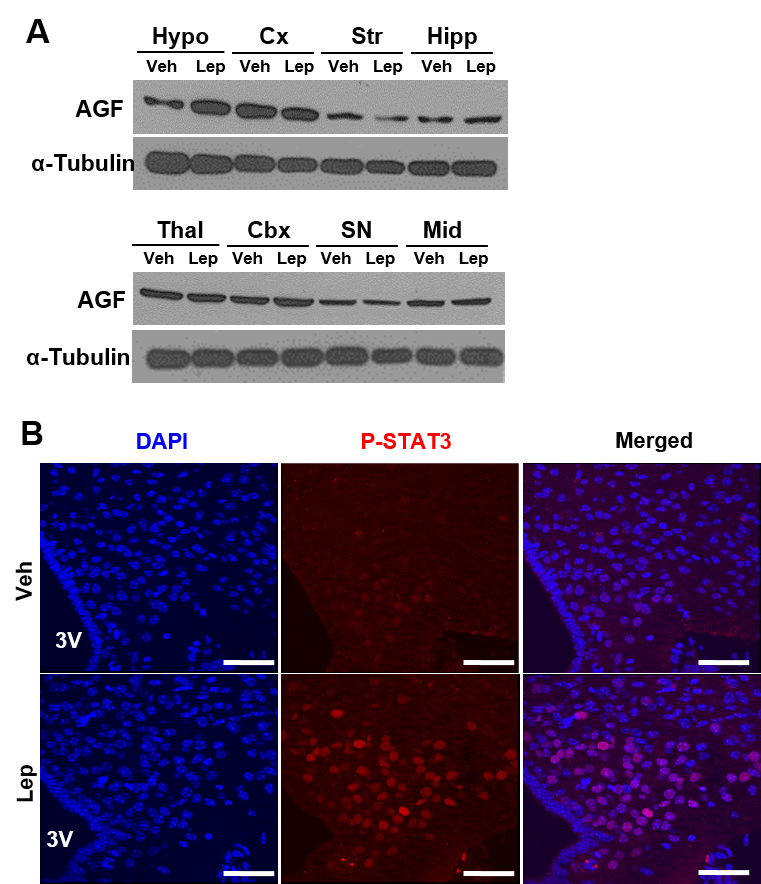

Supplement: Supplementary file 1 [file ijms-22-03443-s001.zip › IJMS Fig S3.tif]

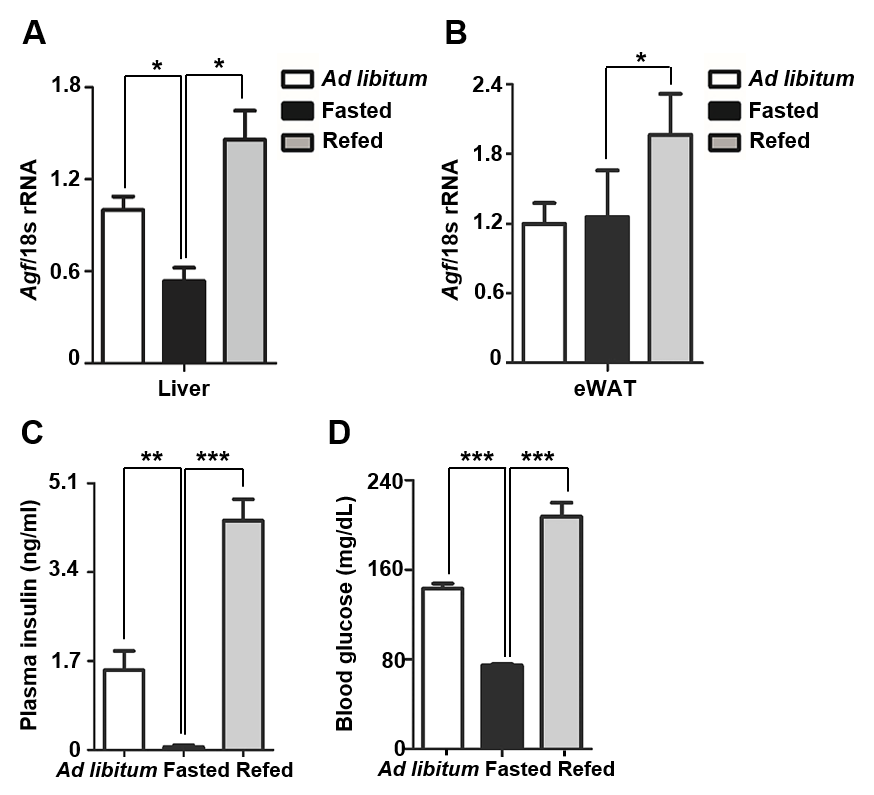

Supplement: Supplementary file 1 [file ijms-22-03443-s001.zip › IJMS FigS1.tif]

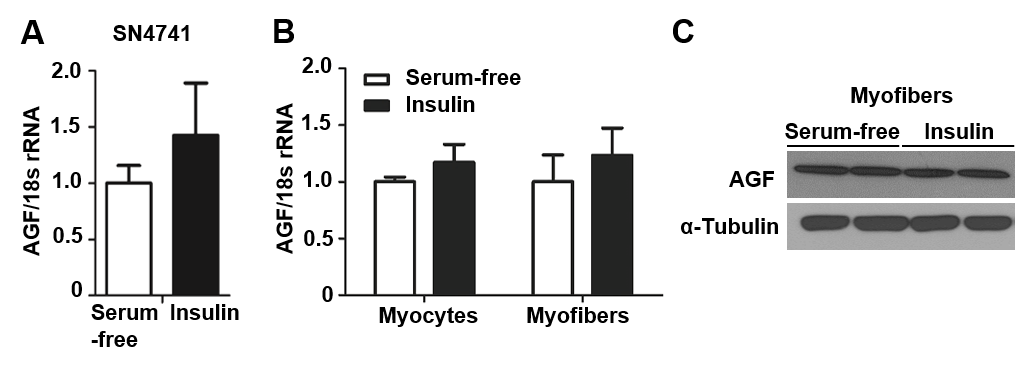

Supplement: Supplementary file 1 [file ijms-22-03443-s001.zip › IJMS FigS2.tif]
